# Adaptive High-Order Sliding Mode Differentiator for Velocity Estimation from Quantized Position Measurements at Ultra-Low Speeds

**DOI:** 10.3390/s26216685

**Published:** 2026-10-22

**Authors:** Haitao Wu, Kuanmin Mao, Fang Fang, Hupo Ouyang, Qihua Xu

**Affiliations:** 1 School of Mechanical Science and Engineering, Huazhong University of Science and Technology, Wuhan 430074, China; wu_seas@126.com (H.W.); maokm@hust.edu.cn (K.M.); 2 Huazhong Institute of Electro-Optics, Wuhan National Laboratory for Optoelectronics, Wuhan 430223, China; ouyanghupo@163.com (H.O.); 17786135770@163.com (Q.X.)

**Keywords:** ultra-low-speed velocity estimation, sliding mode differentiator, quantization noise, adaptive gain, circular grating encoder, position sensor

## Abstract

At ultra-low speeds, the quantization resolution of a position sensor typically exceeds the true angular displacement within one sampling period, so velocity information is almost entirely buried under quantization steps. This paper proposes a quantization-oriented adaptive high-order sliding mode differentiator (Q-AHOSMD). A saturation switching mechanism with the quantization resolution as prior knowledge suppresses high-frequency chattering over quantization plateaus; an event-driven differential velocity estimation scheme of the “update on jump, decay over plateau” type is constructed from quantization jump events; and an adaptive gain law is synthesized from velocity-mismatch feedback. Finite-time convergence of the error system and practical stability under quantization noise are proved, with explicit expressions for all ultimate bounds. Simulations show that, with a sampling period of 1 ms and a true velocity of 0.001°/s, the method achieves a root-mean-square (RMS) velocity estimation error of 0.00036°/s—about 94% lower than the backward difference method—with smooth output and no visible chattering. Requiring no dynamic model and tunable using only sensor specification parameters, the method provides an effective means of extending ultra-precision servo systems into the ultra-low-speed regime.

## 1. Introduction

Precision motion control systems, such as the ultra-low-speed scanning of satellite platform pointing mechanisms [1,2] and the acquisition, link establishment, and stable tracking of space laser communication terminals, require smooth, accurate, and low-latency angular velocity feedback at the load side under extremely low speeds. For example, the pointing mechanism of an Earth observation satellite must perform constant-velocity scanning at approximately 70″/s, and the speed is even lower for Earth–Moon inter-satellite links [1]. When the motion speed falls to the order of 0.001°/s, the actual angular displacement of the position sensor within each sampling period is far smaller than its quantization resolution: the measurement sequence exhibits long unchanged stretches interrupted by occasional jumps, and the effective motion information is almost completely submerged by quantization steps. Reconstructing a continuous, smooth, and low-latency velocity signal from such a heavily quantized position sequence is a core bottleneck in extending ultra-precision servo systems into the ultra-low-speed regime.

Existing research addressing this problem falls roughly into three categories. Filtering-based methods—the backward difference cascaded with a low-pass filter, tracking differentiators, and the like—are model-free and simple to implement. However, under quantized conditions, they cannot escape the rigid trade-off between smoothness and phase lag, and the quantization steps fundamentally limit the resolution floor of difference-based rate measurement [3,4]. Observer-based methods—Luenberger observers [5], sliding mode observers [6,7,8], and dual-sampling-rate observers [2,9]—exploit model prediction to reduce dependence on the high-frequency components of the position signal and offer a higher accuracy ceiling, but their performance depends heavily on model accuracy [10].

The third category is sliding mode differentiator theory. Levant [11] proved that the estimation error of the second-order sliding mode differentiator is proportional to the square root of the noise magnitude and is asymptotically optimal in order and, in a subsequent study, generalized it to arbitrary order within an output-feedback framework [12]. Strict Lyapunov proofs [13], fixed-time convergence [14], adaptive time-varying gains [15,16], varying-gain-exponent schemes [17], and optimal linear adaptive techniques [18] have since been developed, together with low-chattering discrete-time implementations [19,20,21]. However, these studies mainly assume continuous-time models and uniform noise: none incorporates the quantization resolution as known prior knowledge into the design, nor addresses the gain mismatch caused by the sparsity of information during plateau intervals at ultra-low speeds.

To fill this gap, this paper proposes the Q-AHOSMD, which uses only the quantized position sequence from the angle sensor and relies on neither a dynamic model nor auxiliary information. The main contributions are threefold:

(1) An event-driven decaying differential velocity estimation method: the velocity reference is updated from the event interval only when a quantization jump occurs and decays adaptively during plateau periods, converting the sparse quantized position sequence into a continuous velocity reference signal;
(2) An adaptive gain law based on velocity-mismatch feedback: the mismatch between the differential velocity estimate and the differentiator estimate drives a time-varying gain requiring no external prior knowledge, achieving high-gain convergence upon mismatch and low-gain smoothing after tracking; a saturation switch with the quantization resolution as prior knowledge is further introduced to suppress high-frequency chattering on plateaus;
(3) A complete convergence theory: an error model incorporating quantization noise and the boundary layer is established; finite-time convergence in the noise-free case and practical stability in the noisy case are proved; and explicit expressions of all ultimate bounds together with their scaling laws in Δ are given, directly linking each design parameter to a measurable upper bound of the estimation error and forming an engineering tuning guideline requiring only sensor specification parameters.

## 2. Constraints of Quantized Velocity Estimation at Ultra-Low Speeds

This paper takes a 23-bit absolute circular grating as an example for the analysis. The angular position measurement is delivered through a digital interface at a fixed sampling period *T_s_*, corresponding to a quantization resolution of Δ=360°/2^23^≈4.29 × 10^−5^ °. Let the true angular position be *f*(*t*) and the quantized measurement be *f_m_*(*t*)= Δ∙ *round*(*f*(*t*)/Δ); then, the quantization error *η_θ_*≜*f_m_*(*t*) − *f*(*t*) satisfies |*η_θ_*(*t*)|<Δ/2. Moreover, *η_θ_*(*t*) is a signal-dependent error rather than white noise in the usual sense—a property that will be exploited directly in the convergence analysis in Section 4.

Under this constraint, velocity estimation at ultra-low speeds faces the fundamental difficulty of information sparsity. Taking *T_s_* = 1 ms as an example, under the 0.001°/s condition, the actual angular displacement per sampling period is only 10^−6^°, far smaller than Δ; that is, on average, about 43 consecutive sampling periods share exactly the same measurement, and motion information appears only in the form of occasional quantization jumps. This information sparsity is a fundamental constraint that no position-sequence-based velocity estimation method can circumvent.

The minimum resolvable velocity of the backward difference $\hat{\omega }(k)=\left(f_{m}(k)-f_{m}(k-1)\right)/T_{s}$ is Δ/*T_s_*; when the true velocity falls below this value, the output degenerates into a sparse pulse train—zero readings during plateau intervals, interleaved with pulses of amplitude Δ/*T_s_* at quantization jump instants.

Low-pass filters, tracking differentiators, and similar methods can smooth the output, but their common root cause is that the quantization resolution is not fully exploited and the position sequence provides no new information during plateaus; the conflict between smoothness and lag therefore cannot be fundamentally resolved.

Observer-based methods can compensate for the information shortage through model prediction, but their accuracy depends heavily on the model accuracy [10]. At ultra-low speeds, the friction torque is strongly nonlinear and drifts slowly with temperature and lubrication conditions. When the nominal model is mismatched, the model bias is injected directly into the estimate through the correction gains. If the observer bandwidth is increased to suppress the mismatch, the coupling of measurement noise is amplified accordingly, and the observer degenerates into a filter-like method.

The above analysis indicates that under ultra-low-speed conditions, a velocity estimation method is needed that does not rely on an accurate dynamic model and can break through the quantization step constraint.

## 3. Design of the Q-AHOSMD

### 3.1. Fundamentals of the High-Order Sliding Mode Differentiator

For the measured signal *f_m_*(*t*) = *f*(*t*) + *η_θ_*(*t*), assume that the second derivative of the base signal *f*(*t*) is bounded almost everywhere. The second-order sliding mode differentiator proposed by Levant [11], later generalized to arbitrary order in [12] and extended to the time-varying-gain form in [15], takes the form

(1) $$\left\{\begin{array}{c} {\dot{z}}_{0}=-\lambda_{0}\sqrt{L\left(t\right)\left|z_{0}-f\left(t\right)\right|}\mathrm{sign}\left(z_{0}-f\left(t\right)\right)+z_{1}\\ {\dot{z}}_{1}=-\lambda_{1}L\left(t\right)\mathrm{sign}\left(z_{0}-f\left(t\right)\right) \end{array}\right.,$$

where *z*_0_ tracks the position, *z*_1_ estimates the velocity, *λ*_0_ and *λ*_1_ are positive differentiator coefficients chosen according to the classical tuning of [12,15], and *L*(*t*) is a time-varying upper bound of $\left|\ddot{f}(t)\right|$. This form allows the gain to be adjusted in real time as the signal fluctuates violently, but two obstacles arise in engineering practice: first, the sign switching excites high-frequency chattering on quantization plateaus; second, the time-varying bound *L*(*t*) usually has to be obtained from external prior knowledge or auxiliary measurements. The three designs in Section 3.2 address these two obstacles, together with the sparsity of the quantized information itself.

### 3.2. Three Quantization-Oriented Modifications

#### 3.2.1. Saturation Function Switching with the Quantization Resolution as Prior Knowledge

Following the classical boundary layer method of sliding mode control [22], this paper replaces the sign function *sign*(·) with the saturation function *sat*(·):

(2) $$\mathrm{sat}\left(x\right)=\left\{\begin{array}{cc} \mathrm{sign}\left(x\right), & \left|x\right|>\delta \\ \frac{x}{\delta }, & \left|x\right|\leq \delta \end{array}\right.,\delta =\kappa \Delta ,\kappa \geq 1/2,$$

**Figure 1:**
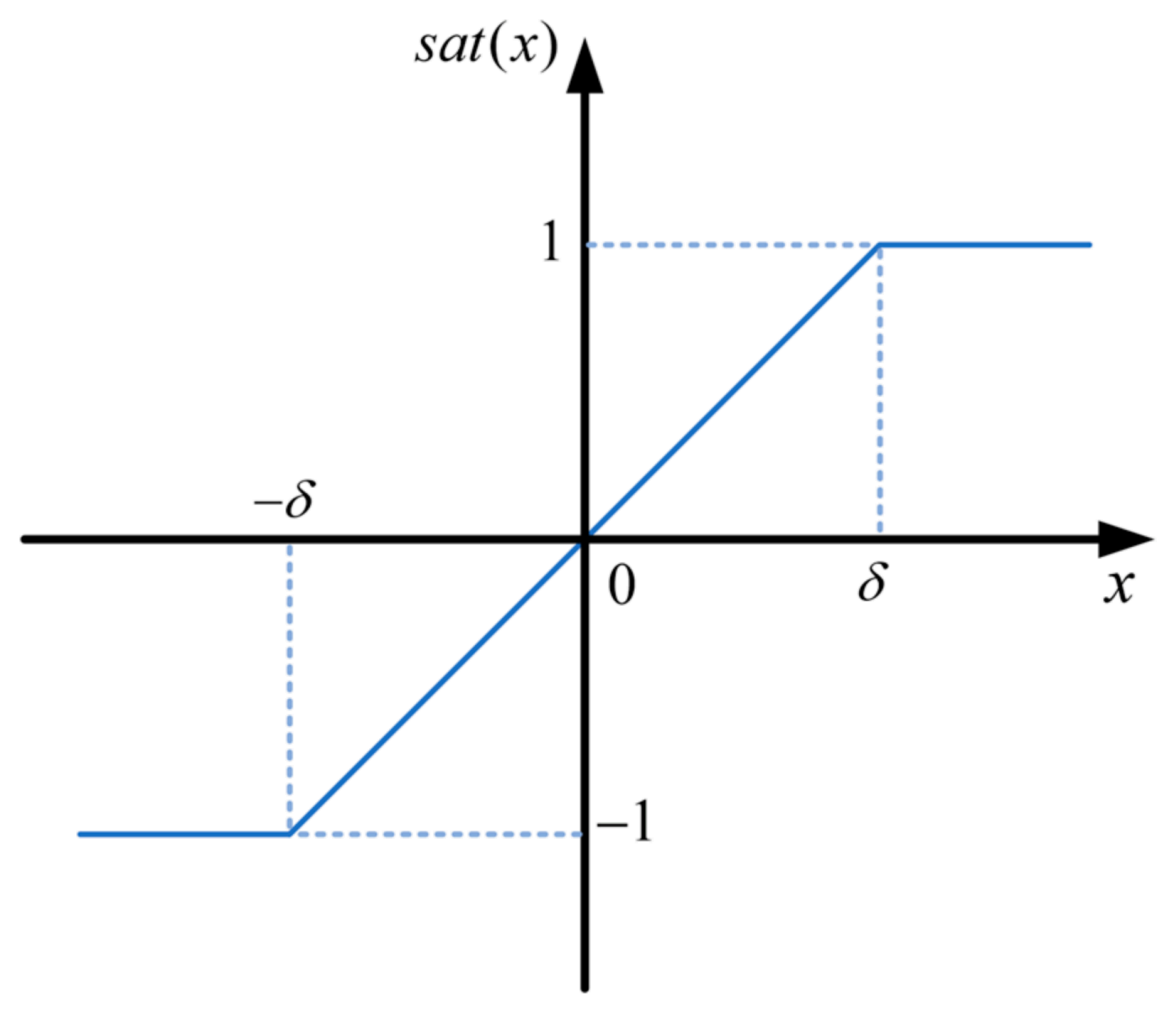
Schematic diagram of the saturation function.

For a sliding mode system, this design preserves the strong feedback of the sliding mode while the tracking error is outside the boundary layer; once the error enters the layer, the feedback becomes proportional to the error, so frequent switching near quantization steps no longer occurs. Section 4 will prove that after the outside layer region is taken as |*e*_0_|>δ+Δ/2, where *e*_0_ = *z*_0_ − *f*(*t*) is the position tracking error, this replacement does not destroy the original convergence mechanism. A schematic diagram of the saturation function is shown in Figure 1.

#### 3.2.2. Decaying Differential Velocity Estimation Based on Quantization Jump Events

Under periodic sampling, the quantized position sequence *f_m_*(*k*) carries new motion information only at jump instants, while the readings remain unchanged during plateau periods. Suppose that the most recent jump occurred at the *k*_0_-th sampling instant, separated from the previous jump by *n* sampling periods, and that the quantized value changed by Δ*f_m_* = *f_m_*(*k*_0_) − *f_m_*(*k*_0_ − *n*). The velocity reference is updated at the jump instant to the average rate between the two quantization events:

(3) $$\omega_{m}\left(k_{0}\right)=\left\{\begin{array}{cc} \frac{\Delta f_{m}}{nT_{s}}, & \left|\Delta f_{m}\right|=\Delta \\ \frac{\Delta f_{m}}{T_{s}}, & \left|\Delta f_{m}\right|>\Delta \end{array}\right.,$$

**Figure 2:**
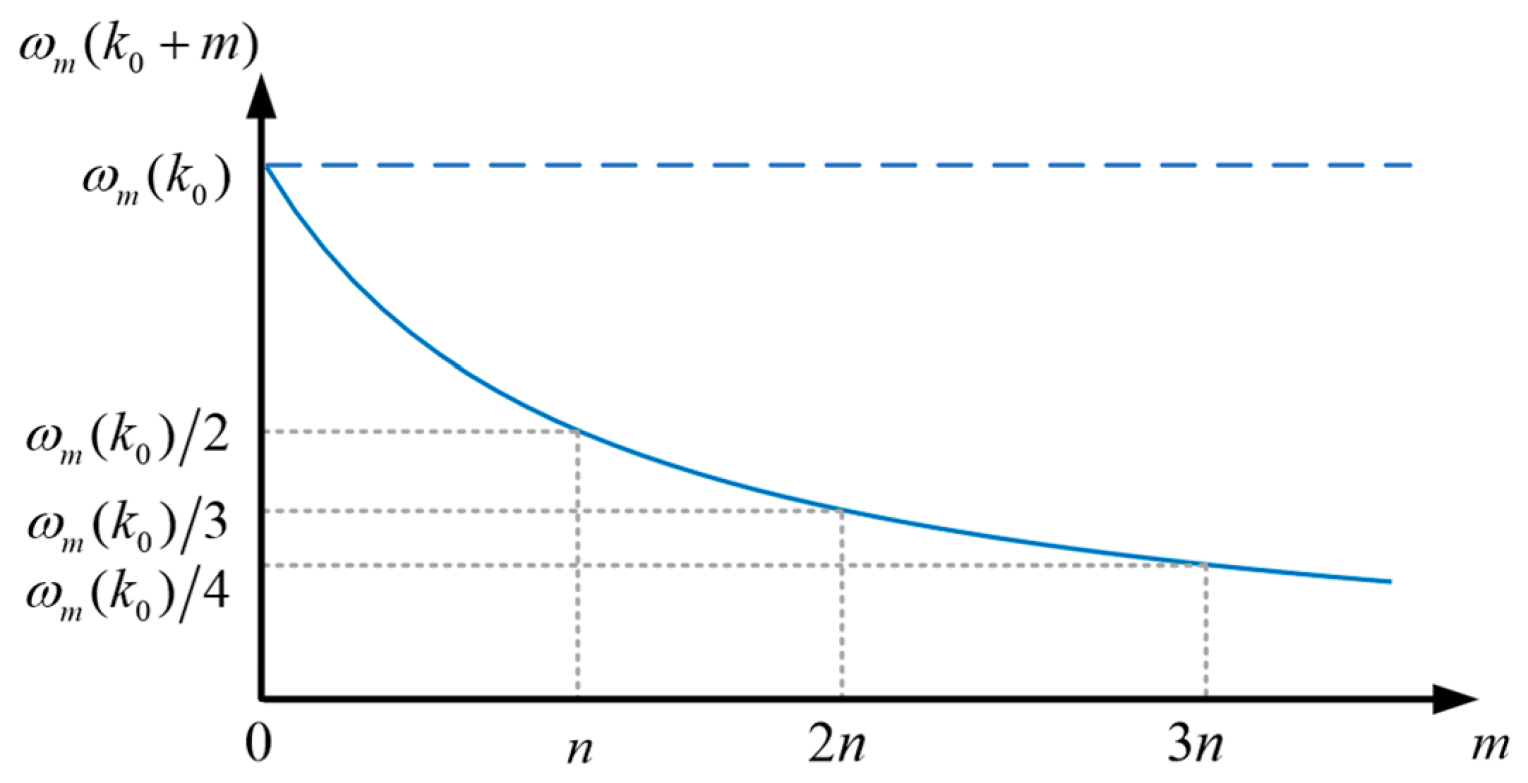
Schematic diagram of the plateau decay mechanism.

The update law (cf. Equation (3)) follows the same principle as the M/T-type velocity measurement for incremental encoders [4], where the speed is estimated from the elapsed time between two position increments. It should be noted that this updated value has a clear physical meaning only at the jump instant. If *m* sampling periods have elapsed after a jump without the next jump arriving, this observation itself implies velocity information: the true speed has most likely fallen below Δ/(*mT_s_*) and keeps decreasing as *m* grows. If *ω_m_* stayed unchanged at the last jump rate, it would continuously feed a false mismatch signal into the adaptive gain law (cf. Equation (5)) below, misleading the gain to keep rising. A plateau decay mechanism is therefore introduced:

(4) $$\omega_{m}\left(k_{0}+m\right)=\omega_{m}\left(k_{0}\right)\cdot d(m),d(m)=\frac{\mathrm{\gamma n}}{\mathrm{\gamma n}+m},$$

where *m* is the cumulative number of sampling steps in the current plateau, *n* is the total number of steps in the previous plateau, and *γ* > 0 is the decay coefficient—the larger *γ* is, the slower the decay; *γ* = 1 in this paper. The decay curve is shown in Figure 2.

**Figure 3:**
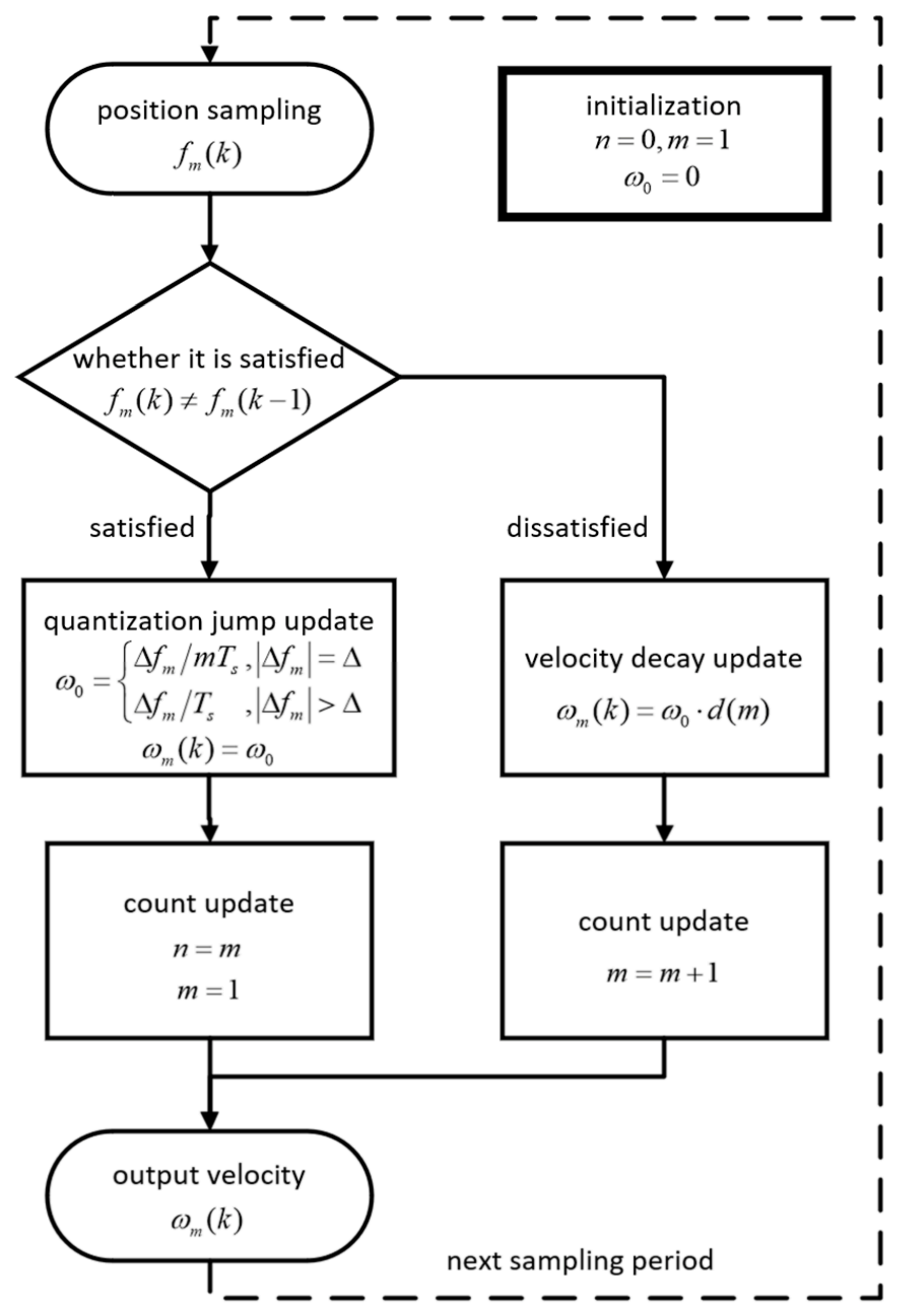
Flowchart of the decaying differential velocity estimation based on quantization jump events.

The velocity estimation procedure is illustrated in Figure 3: the rate is updated when a quantization jump occurs in the position, and it keeps decaying on the basis of the latest jump rate during plateaus in which the position does not change.

#### 3.2.3. Adaptive Gain Law Based on Velocity-Mismatch Feedback

The difference *e_m_*_1_ = *z*_1_ − *ω_m_* between the velocity estimate *z*_1_ of the differentiator and the velocity estimate given by the above event-based differential estimation is a direct measure of the current tracking mismatch, i.e., of the required convergence strength: a large mismatch means that the differentiator has not yet caught up with the true motion and a large gain is needed for fast convergence; when the mismatch vanishes, the tracking is good and the gain should become small so as not to amplify noise. Accordingly, the mismatch is normalized by *pT_s_* to construct the time-varying gain

(5) $$L\left(t\right)=\max\left\{L_{\min},\frac{\left|e_{m1}\right|}{pT_{s}}\right\},$$

where *p* > 0 is the mismatch coefficient and *L_min_* > 0 is the gain floor.

### 3.3. Complete Implementation

The following three steps are executed in sequence in each sampling period:

Step 1 (velocity estimation): compute *ω_m_* according to the jump-event update and the plateau decay (cf. Equations (3) and (4));

Step 2 (gain computation):

(6) $$L\left(t\right)=\max\left\{L_{\min},\frac{\left|e_{m1}\right|}{pT_{s}}\right\},$$

Step 3 (differentiator update):

(7) $$\left\{\begin{array}{c} {\dot{z}}_{0}=-\lambda_{0}\sqrt{L\left(t\right)\left|e_{m0}\right|}\mathrm{sat}\left(e_{m0}\right)+z_{1}\\ {\dot{z}}_{1}=-\lambda_{1}L\left(t\right)\mathrm{sat}\left(e_{m0}\right) \end{array}\right.,$$

where *e_m_*_0_ = *z*_0_ − *f_m_*.

**Figure 4:**
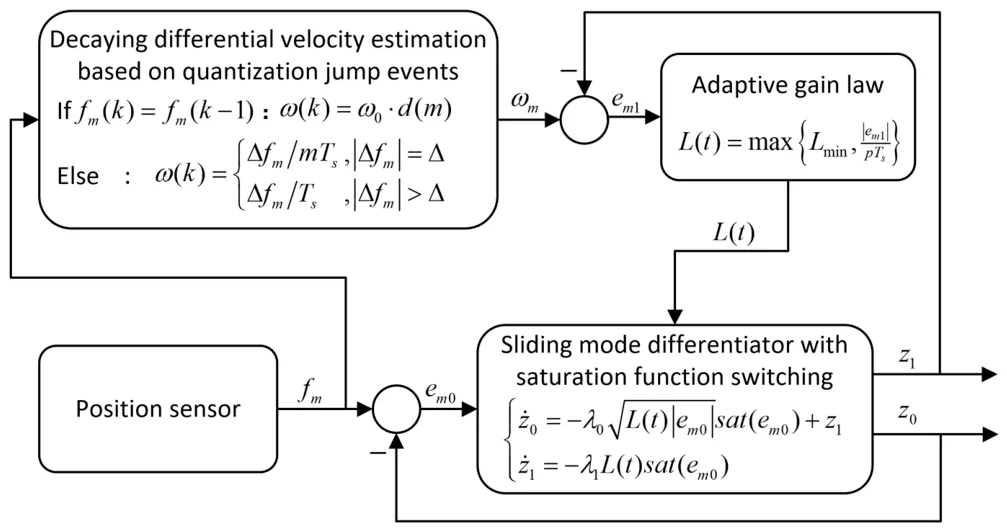
Block diagram of the Q-AHOSMD.

The overall structure of the proposed Q-AHOSMD is illustrated in Figure 4.

## 4. Convergence Analysis and Parameter Tuning

This section establishes the complete convergence theory of the Q-AHOSMD. The proof proceeds as follows: finite-time convergence is first proved in the ideal case without noise and without the boundary layer (Theorem 1); it is then proved that the effect of the quantization noise and the sat boundary layer is merely to inflate each dividing line outward by a bounded margin, with all convergence mechanisms preserved one by one (Theorem 2), and explicit expressions of the ultimate bounds are given.

### 4.1. Assumptions, Notation, and Error Model

The following variables are defined. Tracking error and velocity error:

(8) $$e_{0}=z_{0}-f,e_{1}=z_{1}-\dot{f},$$

Computable innovation errors:

*e_m_*_0_ = *z*_0_ − *f_m_* = *e*_0_ − *η_θ_*, *e_m_*_1_ = *z*_1_ − *ω_m_* = *e*_1_ − *η_ω_*, (9)

Normalization constants and gain notation:

(10) $$c_{0}=\frac{\lambda_{0}}{\sqrt{pT_{s}}},c_{1}=\frac{\lambda_{1}}{pT_{s}},G=\max\left\{\bar{g},\left|e_{m1}\right|\right\},\rho ={\left(\bar{g}-\left|e_{m1}\right|\right)}_{+},$$

**Assumption** **1** (signal boundedness)**.** *The base signal f*(*t*) *is twice differentiable, and there exists a constant M such that* $\left|\ddot{f}(t)\right|\leq M$ *almost everywhere.*

**Assumption** **2** (measurement error boundedness)**.** *The position quantization error satisfies η_θ_*(*t*) = *f_m_*(*t*) − *f*(*t*)*,* |*η_θ_*(*t*)|<Δ/2; *the velocity measurement error satisfies* $\eta_{\omega }(t)=\omega_{m}(t)-\dot{f}(t)$ *,* |*η_ω_*(*t*)|≤Δ/*T_s_*. *The latter upper bound corresponds to the extreme case in which one quantization step is accumulated within a single sampling period.*

**Assumption** **3** (gain-floor matching)**.** $\bar{g}=pT_{s}L_{\min}\leq M/c_{1}$.

The active branch (*G* = |*e_m_*_1_|) and the truncated branch ($G=\bar{g}$) of G together cover all values of the gain *L*.

Error dynamics: outside the sat boundary layer (*sat*(*e_m_*_0_) = *sign*(*e_m_*_0_)), one obtains

(11) $$\left\{\begin{array}{c} {\dot{e}}_{0}=e_{1}-c_{0}\sqrt{G\left|e_{m0}\right|}\mathrm{sign}\left(e_{m0}\right)\\ {\dot{e}}_{1}=-c_{1}\mathrm{Gsign}\left(e_{m0}\right)-\ddot{f}(t) \end{array}\right.,$$

### 4.2. Noise-Free Case: Finite-Time Convergence

Let *η_θ_* = *η_ω_* = 0 and δ=0; then, *e_m_*_0_ = *e*_0_, *e_m_*_1_ = *e*_1_, and $G=\max\left\{\bar{g},\left|e_{1}\right|\right\}$.

(12) $$D^{+}V\leq c_{1}\rho +M-c_{0}c_{1}\sqrt{G\left|e_{0}\right|},$$

**Lemma** **1.** *Let V* = *c*_1_|*e*_0_| + |*e*_1_|*. Along the trajectories of Equation (11),*

*In particular, when *$\left|e_{1}\right|>\bar{g}$ *(the active branch), the first term cancels identically, and *$D^{+}V\leq M-c_{0}c_{1}\sqrt{\left|e_{1}\right|\left|e_{0}\right|}$.

**Proof** **of** **Lemma** **1.** From $D^{+}\left|x\right|=\dot{x}\cdot \mathrm{sign}\left(x\right)$, $\left|\ddot{f}(t)\right|\leq M$, and |*e*_1_| − *G* ≤ *ρ*, one obtains: $D^{+}V=c_{1}\mathrm{sign}\left(e_{0}e_{1}\right)\left(\left|e_{1}\right|-G\right)-c_{0}c_{1}\sqrt{G\left|e_{0}\right|}-\ddot{f}\left(t\right)\mathrm{sign}\left(e_{1}\right)\leq c_{1}\rho +M-c_{0}c_{1}\sqrt{G\left|e_{0}\right|}$.

When $\left|e_{1}\right|>\bar{g}$, |*e*_1_| = *G*, and hence $D^{+}V\leq M-c_{0}c_{1}\sqrt{\left|e_{1}\right|\left|e_{0}\right|}$. □

**Theorem** **1** (noise-free finite-time convergence)**.** *Suppose that Assumptions 1 and 3 hold, η_θ_* = *η_ω_* = 0*,* δ=0*, and the parameters are arbitrary positive. Let* $H={\left(2M/\left(c_{0}c_{1}\right)\right)}^{2}$*. Then, the error system enters the following region within finite time and remains there permanently:*

(13) $$\left|e_{1}\right|\leq S_{1}≜\frac{2M}{c_{1}}+\frac{3M}{c_{0}^{2}},\left|e_{0}\right|\leq S_{0}≜\max\left\{\frac{4\bar{g}}{{c_{0}}^{2}},\frac{S_{1}}{{c_{0}}^{2}}\right\},$$

**Proof** **of** **Theorem** **1.** The phase plane is partitioned by |*e*_0_*e*_1_| = *H* (the hyperbolic bound) and |*e*_1_| = 2*M*/*c*_1_ (the strip bound):

Zone A (|*e*_0_*e*_1_| ≥ *H*): by Lemma 1, $D^{+}V\leq M-c_{0}c_{1}\sqrt{\left|e_{0}\right|\left|e_{1}\right|}\leq -M<0$; V decreases strictly at a rate no less than *M*, and the dwell time does not exceed *V*(0)/*M*.

Zone B (|*e*_0_*e*_1_| < *H* and |*e*_1_| > 2*M*/*c*_1_): geometrically, $\left|e_{0}\right|<H/\left|e_{1}\right|\leq 2M/\left({c_{0}}^{2}c_{1}\right)$.

B1 (e_0_e_1_ > 0): $D^{+}\left|e_{1}\right|=-c_{1}\left|e_{1}\right|-\ddot{f}(t)\mathrm{sign}\left(e_{1}\right)<-M$; |*e*_1_| decreases linearly at a rate greater than *M* until entering |*e*_1_| ≤ 2*M*/*c*_1_.

B2 (e_0_e_1_ < 0): $D^{+}\left|e_{0}\right|=\left|e_{1}\right|\mathrm{sign}\left(e_{0}e_{1}\right)-c_{0}\sqrt{\left|e_{1}\right|\left|e_{0}\right|}\leq -\left|e_{1}\right|\leq -2M/c_{1}$, so e_0_ must cross the *e*_0_ = 0 axis and enter B1 within $\tau \leq 1/{c_{0}}^{2}$.

Meanwhile, $D^{+}\left|e_{1}\right|=c_{1}\left|e_{1}\right|-\ddot{f}\left(t\right)\mathrm{sign}\left(e_{1}\right)\leq -c_{1}D^{+}\left|e_{0}\right|+M$; integration shows that the increment of |*e*_1_| does not exceed $3M/{c_{0}}^{2}$. This means that after entering B2, each crossing starts from the boundary |*e*_1_| = 2*M*/*c*_1_, and the peak value therefore does not exceed *S*_1_.

Truncated branch ($\left|e_{1}\right|\leq \bar{g}$): the trajectory is already inside the ultimate convergence region.

Once |*e*_1_| ≤ *S*_1_ holds, further analysis is as follows.

Active branch: *G* = |*e*_1_|, $\left|e_{1}\right|>\bar{g}$, so $D^{+}\left|e_{0}\right|=\mathrm{sign}\left(e_{0}\right)e_{1}-c_{0}\sqrt{G\left|e_{0}\right|}\leq \left|e_{1}\right|-c_{0}\sqrt{\left|e_{1}\right|\left|e_{0}\right|}$; thus, if $\left|e_{0}\right|>\left|e_{1}\right|/{c_{0}}^{2}$, then *D*^+^|*e*_0_| < 0 and |*e*_0_| decreases strictly;

Truncated branch: $G=\bar{g}$, |*e*_1_| ≤ *g*, so $D^{+}\left|e_{0}\right|=\mathrm{sign}\left(e_{0}\right)e_{1}-c_{0}\sqrt{G\left|e_{0}\right|}\leq \left|e_{1}\right|-c_{0}\sqrt{G\left|e_{0}\right|}<\bar{g}-c_{0}\sqrt{\bar{g}\left|e_{0}\right|}$; thus, if $\left|e_{0}\right|>4\bar{g}/{c_{0}}^{2}$, then *D*^+^|*e*_0_| < 0 and |*e*_0_| decreases strictly.

Taking the maximum of the two branches as the upper bound of |*e*_0_| gives $\left|e_{0}\right|\leq S_{0}=\max\{4\bar{g}/{c_{0}}^{2},S_{1}/{c_{0}}^{2}\}$. □

### 4.3. Quantization Noise and the Sat Boundary Layer: Bounded Inflation of the Convergence Region

Assumption 2 is now enabled, with δ=*κ*Δ (*κ* ≥ 0.5). The region outside the sat boundary layer is taken as |*e*_0_|>δ+Δ/2; there, |*e_m_*_0_|>δ, and sat(*e_m_*_0_) = sign(*e_m_*_0_) = sign(*e*_0_), i.e., saturation consistency and sign consistency hold simultaneously.

**Lemma** **2.** *Outside the sat boundary layer, along the trajectories of Equation (11), on the active branch,*

(14) $$D^{+}V\leq -c_{0}c_{1}\sqrt{G\left|e_{m0}\right|}+c_{1}\beta ,\beta ≜\frac{\Delta }{T_{s}}+\frac{M}{c_{1}},$$

**Proof** **of** **Lemma** **2.** As in Lemma 1, one obtains: $D^{+}V=c_{1}\mathrm{sign}\left(e_{0}e_{1}\right)\left(\left|e_{1}\right|-G\right)-c_{0}c_{1}\sqrt{G\left|e_{m0}\right|}-\ddot{f}\left(t\right)\mathrm{sign}\left(e_{1}\right)$.

When $\left|e_{m1}\right|=\left|e_{1}-\eta_{\omega }\right|\geq \bar{g}$, the triangle inequality gives |*e*_1_| − *G* ∈ [−Δ/*T_s_*,Δ/*T_s_*], and hence $D^{+}V\leq -c_{0}c_{1}\sqrt{G\left|e_{m0}\right|}+c_{1}\beta$ with *β*≜Δ/*T_s_* + *M*/*c*_1_. The only difference from Lemma 1 is that the identity cancellation degenerates into the triangle inequality ||*e*_1_| − |*e_m_*_1_||≤Δ/*T_s_*: the entire effect of the measurement error is to raise the dissipation balance baseline from *M*/*c*_1_ to *β*. □

**Theorem** **2** (practical stability and explicit ultimate bounds)**.** *Suppose that Assumptions 1–3 hold, the parameters are arbitrary positive, and κ ≥ 1/2. Then*

(15) $$\underset{t\to \infty }{\mathrm{lim sup}}\left|e_{1}\left(t\right)\right|\leq S_{1}≜4\beta +\frac{3c_{1}\beta }{c_{0}^{2}}+c_{1}\left(\delta +\frac{\Delta }{2}\right),$$

(16) $$\underset{t\to \infty }{\mathrm{lim sup}}\left|e_{0}\left(t\right)\right|\leq S_{0}≜\max\left\{\frac{{S_{1}}^{2}}{{c_{0}}^{2}\left(S_{1}-\frac{\Delta }{T_{s}}\right)},\frac{2\beta }{{c_{0}}^{2}},\frac{{\left(\frac{\Delta }{T_{s}}+\bar{g}\right)}^{2}}{{c_{0}}^{2}\bar{g}}\right\}+\left(\kappa +\frac{1}{2}\right)\Delta ,$$

**Proof** **of** **Theorem** **2.** Following the partitioning and discussion of Theorem 1, each dividing line is replaced by its inflated version (the hyperbolic bound $H\to H_{V}={\left(2\beta /c_{0}\right)}^{2}$, and the strip bound 2*M*/*c*_1_ → 2*β*), and the preservation of the mechanism is verified zone by zone:

Zone A (*G*|*e*_0_| > *H_V_*): outside the sat boundary layer, |*e_m_*_0_| ≥ |*e*_0_|/2 (*κ* ≥ 1/2); by Lemma 2, $D^{+}V<-c_{0}c_{1}\sqrt{H_{V}/2}+c_{1}\beta =-\left(\sqrt{2}-1\right)c_{1}\beta <0$;

Zone B1 (*G*|*e*_0_| ≤ *H_V_*, |*e*_1_| > 2*β*, *e*_0_*e*_1_ > 0): *D*^+^|*e*_1_| ≤ −*c*_1_(|*e*_1_| − *β*) < −*c*_1_*β*; the velocity is pressed linearly toward the strip bound at a rate greater than *c*_1_*β*;

Zone B2 (*G*|*e*_0_| ≤ *H_V_*, |*e*_1_| > 2*β*, *e*_0_*e*_1_ < 0): the condition for entering Zone B2 immediately gives $\left|e_{0}\right|\leq H_{V}/\left|e_{1}\right|\leq 2\beta /{c_{0}}^{2}$; after entering Zone B2, the trajectory leaves within $\tau <1/{c_{0}}^{2}$; from *D*^+^|*e*_1_| ≤ −*c*_1_*D*^+^|*e*_0_| + *c*_1_*β*, integration gives an increment of |*e*_1_| no greater than $3c_{1}\beta /{c_{0}}^{2}$; with a conservative estimate in the extreme case, Equation (15) follows;

Truncated branch ($G=\bar{g}$): $\left|e_{1}\right|\leq \bar{g}+\Delta /T_{s}\leq \beta$ (Assumption 3), already inside the strip.

After |*e*_1_| ≤ *S*_1_ holds, $D^{+}\left|e_{0}\right|\leq \left|e_{1}\right|-c_{0}\sqrt{G\left(\left|e_{0}\right|-\Delta /2\right)}$; further analysis is as follows.

Active branch: *G* = |*e_m_*_1_| = |*e*_1_ − *η_ω_*| ≥ |*e*_1_|−Δ/*T_s_* and $\left|e_{m1}\right|\geq \bar{g}$; when |*e*_1_| > 2*β*, the condition $\left|e_{0}\right|-\Delta /2\geq {S_{1}}^{2}/\left({c_{0}}^{2}\left(S_{1}-\Delta /T_{s}\right)\right)$ guarantees *D*^+^|*e*_0_| < 0; when |*e*_1_| ≤ 2*β*, the condition $\left|e_{0}\right|>{\left(2\beta \right)}^{2}/\left({c_{0}}^{2}\left(2\beta -\Delta /T_{s}\right)\right)\geq 2\beta /{c_{0}}^{2}$ guarantees *D*^+^|*e*_0_| < 0;

Truncated branch: $G=\bar{g}$, $\left|e_{m1}\right|\leq \left|e_{1}\right|+\Delta /T_{s}\leq \bar{g}$; the condition $\left|e_{0}\right|-\Delta /2\geq {\left(\Delta /T_{s}+\bar{g}\right)}^{2}/\left({c_{0}}^{2}\bar{g}\right)$ guarantees *D*^+^|*e*_0_| < 0.

Taking the maximum of the three terms yields the ultimate bound (cf. Equation (16)) of the position error *e*_0_, which completes the proof. □

**Figure 5:**
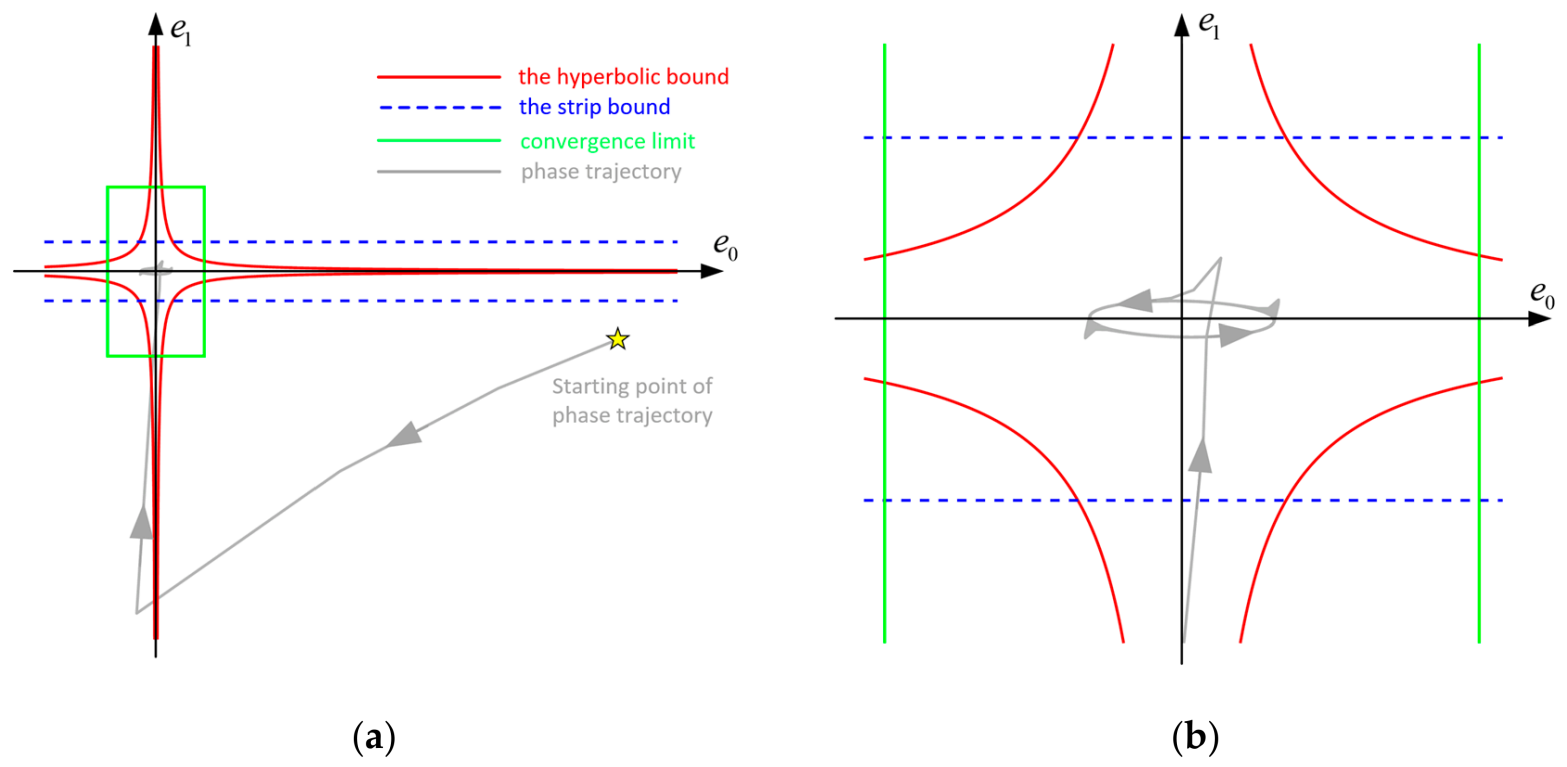
Phase-plane convergence diagram of the Q-AHOSMD: (**a**) dividing lines and phase trajectories; (**b**) limit cycle of the phase trajectory.

As shown in Figure 5, according to the foregoing definitions and the corresponding proofs, the convergence process of a typical Q-AHOSMD in the (*e*_0_, *e*_1_) phase plane is illustrated, together with the hyperbolic bound, the strip bound, and the ultimate convergence bound. It can be seen that the phase trajectory eventually converges to the ultimate bound within finite time and forms a limit cycle near the origin.

### 4.4. Parameter Tuning Guidelines

The parameters of the Q-AHOSMD are tuned in the order “gain coefficients *λ*_0_, *λ*_1_ → sampling period *T_s_* → mismatch coefficient *p* and boundary layer coefficient *κ* → gain floor *L_min_*”.

(1) Gain coefficients *λ*_0_ and *λ*_1_. These are selected according to the classical HOSMD values [12,15]; this paper uses *λ*_0_ = 1.1 and *λ*_1_ = 1.5. This pair of coefficients determines the ratio between the convergence rate and the ultimate bound; fine-tuning around the classical values suffices, and they are not sensitive parameters.

(2) Sampling period *T_s_*. *T_s_* is chosen from the options given by the hardware gears of the sensor and the controller. The choice of *T_s_* must balance quantization noise against sampling lag. Collecting and classifying the *T_s_*-dependent parts of the position and velocity ultimate bounds gives

(17) $$S_{0d}=S_{0}+T_{s}\max\left|\dot{f}\right|,$$

(18) $$S_{1d}=S_{1}+MT_{s}=\underset{\mathrm{noise}\;\mathrm{channel}}{\underbrace{\left(4+\frac{3\lambda_{1}}{{\lambda_{0}}^{2}}\right)\frac{\Delta }{T_{s}}}}+\underset{\mathrm{signal}\;\mathrm{channel}}{\underbrace{\left(\frac{4}{\lambda_{1}}+\frac{3}{{\lambda_{0}}^{2}}\right)\mathrm{Mp}T_{s}}}+\underset{\mathrm{saturation}\;\mathrm{layer}}{\underbrace{\frac{\lambda_{1}\left(\kappa +1/2\right)\Delta }{pT_{s}}}}+\underset{\mathrm{measurement}\;\mathrm{delay}}{\underbrace{MT_{s}}},$$

where the four terms on the right-hand side correspond, in order, to the noise channel (proportional to 1/*T_s_*), the signal channel (proportional to *T_s_*), the saturation layer (proportional to 1/*T_s_*), and the measurement delay (proportional to *T_s_*). It can be seen that *S*_0*d*_ increases monotonically with *T_s_*—the smaller *T_s_* is, the better in the position direction, so it only provides an upper-bound constraint for model selection.

Variation in *S*_1*d*_ with *T_s_*: differentiating *S*_1*d*_ gives

(19) $${S^{\prime }}_{1d}=-\left(4+\frac{3\lambda_{1}}{{\lambda_{0}}^{2}}+\frac{\lambda_{1}\left(\kappa +1/2\right)}{p}\right)\frac{\Delta }{{T_{s}}^{2}}+\left(\frac{4}{\lambda_{1}}+\frac{3}{{\lambda_{0}}^{2}}\right)\mathrm{Mp}+M,$$

Setting the derivative to zero yields the minimum point (the second derivative is always positive, so this is indeed a minimum):

(20) $$T_{s}^{*}=\sqrt{\frac{\lambda_{1}\Delta \left[p\left(4{\lambda_{0}}^{2}+3\lambda_{1}\right)+{\lambda_{0}}^{2}\lambda_{1}\left(\kappa +1/2\right)\right]}{\mathrm{pM}\left[{\lambda_{0}}^{2}\lambda_{1}+p\left(4{\lambda_{0}}^{2}+3\lambda_{1}\right)\right]}},$$

In terms of the variation pattern: when $T_{s}<T_{s}^{*}$, the error grows steeply mainly as 1/*T_s_*, whereas when $T_{s}>T_{s}^{*},$ it grows only slowly, as *T_s_*. Therefore, a larger value within the available range is preferable.

(3) Mismatch coefficient *p*. The mismatch coefficient *p* is introduced in the adaptive gain *L* and is the sensitivity coefficient linking the quantization mismatch to the sliding mode gain. According to the Euler discretization requirement, *c*_1_*T_s_* = *λ*_1_/*p* ≤ 1, i.e., *p* ≥ *λ*_1_; the recommended choice is *p* ∈ [*λ*_1_,3*λ*_1_].

(4) Gain floor *L_min_*. The main role of *L_min_* is to prevent the velocity error *e*_1_ from stagnating and failing to converge further when the error crosses *e*_0_ = 0. Therefore, its value must satisfy *L_min_* > 0, but an excessively large Lmin would degrade the differentiator into a fixed-gain structure and lose the adaptive capability.

(5) Boundary layer coefficient *κ*. The layer thickness of the saturation function is δ = *κ*Δ, and *κ* determines the extent to which the quantization error is covered by the linear region. The recommended range is *κ* ∈ [1/2,3]. Increasing *κ* slows the dynamic convergence and enlarges the error but yields a smoother output; decreasing *κ* accelerates the convergence and reduces the error but makes the system more sensitive to noise.

**Remark** **1** **.** *In practical digital readouts, the reading may toggle between two adjacent codes near a quantization boundary (last-digit noise). This bounded perturbation (within one quantization step) is covered by the analysis in Section 4, and its influence on ω_m_ is transient and self-correcting. If significant last-digit noise is expected, a larger κ within the recommended range yields a smoother output, at the cost of a moderately enlarged ultimate bound (cf. Equation (16)).*

## 5. Simulation Results

Since the proposed Q-AHOSMD is essentially a signal processing method that operates directly on the quantized position sequence, its performance is independent of any specific physical platform and can be exactly characterized by numerical simulation. In addition, the true velocity required as the error reference is difficult to obtain in a physical experiment at ultra-low speeds, whereas it is exactly known in simulation. Simulations are therefore conducted for the application scenario in which only an angular position sensor is used to obtain the angular velocity under ultra-low-speed conditions. Under 23-bit quantization and a sampling frequency of 1000 Hz, the velocity estimation performance of three methods—the backward difference (BD), the classical HOSMD, and the proposed Q-AHOSMD—is compared. The parameters used by the HOSMD and the Q-AHOSMD are listed in Table 1.

Considering different working conditions, the velocity estimation is simulated for ultra-low-speed constant-velocity motion and for sinusoidal motion; to verify the generality of the method, a sinusoidal-motion estimation simulation at a relatively high speed is also conducted.

### 5.1. Ultra-Low-Speed Constant-Velocity Motion

**Figure 6:**
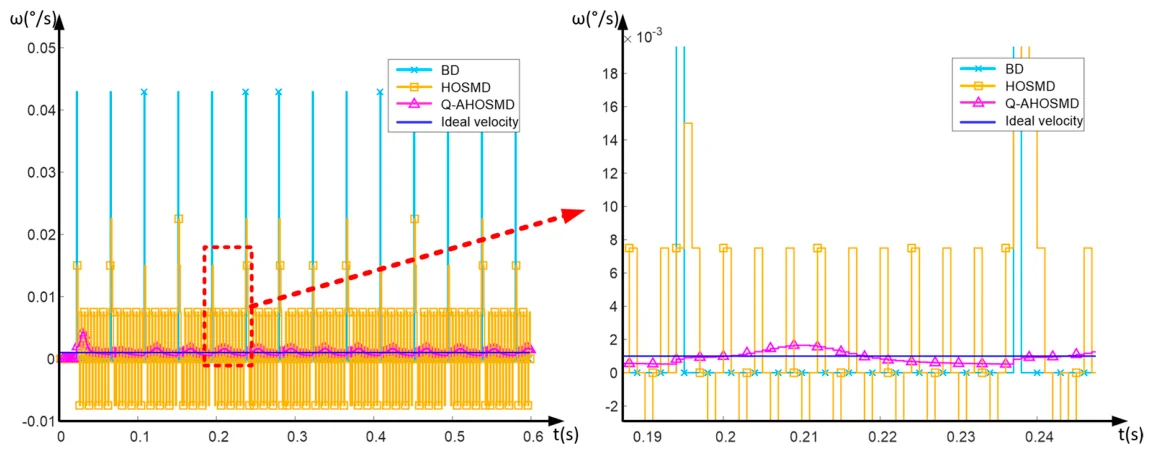
Velocity estimation curves under the ultra-low-speed constant-velocity condition.

Under the ultra-low-speed constant-velocity condition of 0.001°/s, the velocity estimation curves and the error statistics are shown in Figure 6 and Table 2.

It can be seen that under the ultra-low-speed constant-velocity condition, the BD exhibits isolated spikes at quantization jump instants and outputs near zero during plateaus, failing to form a continuous velocity feedback. To guarantee accuracy, the HOSMD requires *L* to be set very small, and obvious chattering arises under the influence of the sign function. The Q-AHOSMD output is smooth and tracks the true velocity effectively, with a velocity estimation error of 0.00036°/s, about 94% lower than those of both the HOSMD (0.0064°/s) and the BD (0.0066°/s).

It should be noted that the HOSMD faces an inherent trade-off between accuracy and chattering under this condition: increasing L aggravates sign-switching chattering near quantization steps, while decreasing it slows convergence and increases tracking lag. The setting of *L* for the HOSMD in Table 2 is a compromise between dynamics and accuracy.

### 5.2. Ultra-Low-Speed Sinusoidal Motion

**Figure 7:**
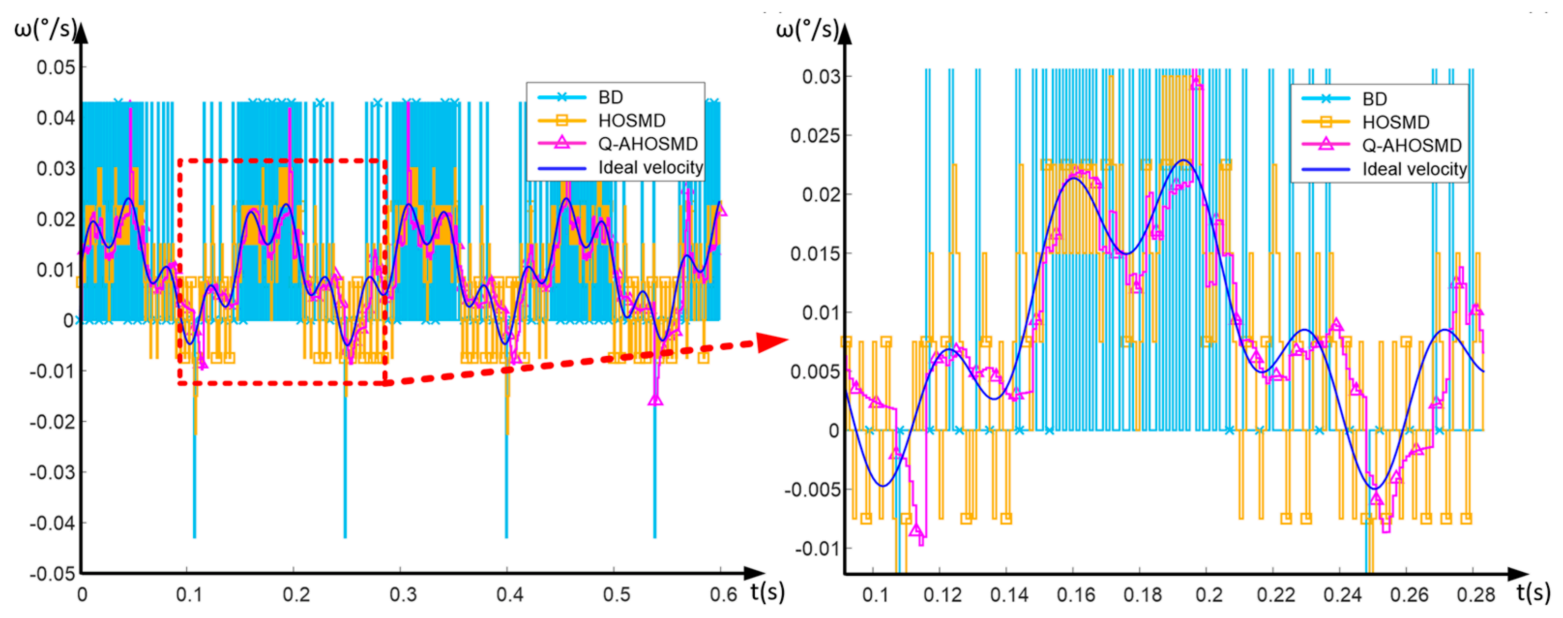
Velocity estimation curves under the ultra-low-speed sinusoidal condition.

With the true angular position input *f*(*t*) = (0.01 + 0.01sin(2*π* × 7*t*) + 0.005sin(2*π* × 27*t*))°, the velocity estimation curves and the error statistics are shown in Figure 7 and Table 3.

It can be seen that under the ultra-low-speed sinusoidal tracking condition, the BD still produces spikes only at quantization jumps; the HOSMD output reflects the rough sinusoidal profile but is superimposed with obvious chattering. The Q-AHOSMD fluctuates slightly around the true velocity without visible chattering, and its RMS error (0.0037°/s) is about 47% lower than that of the HOSMD (0.007°/s) and about 78% lower than that of the BD (0.017°/s).

### 5.3. Higher-Speed Sinusoidal Motion

**Figure 8:**
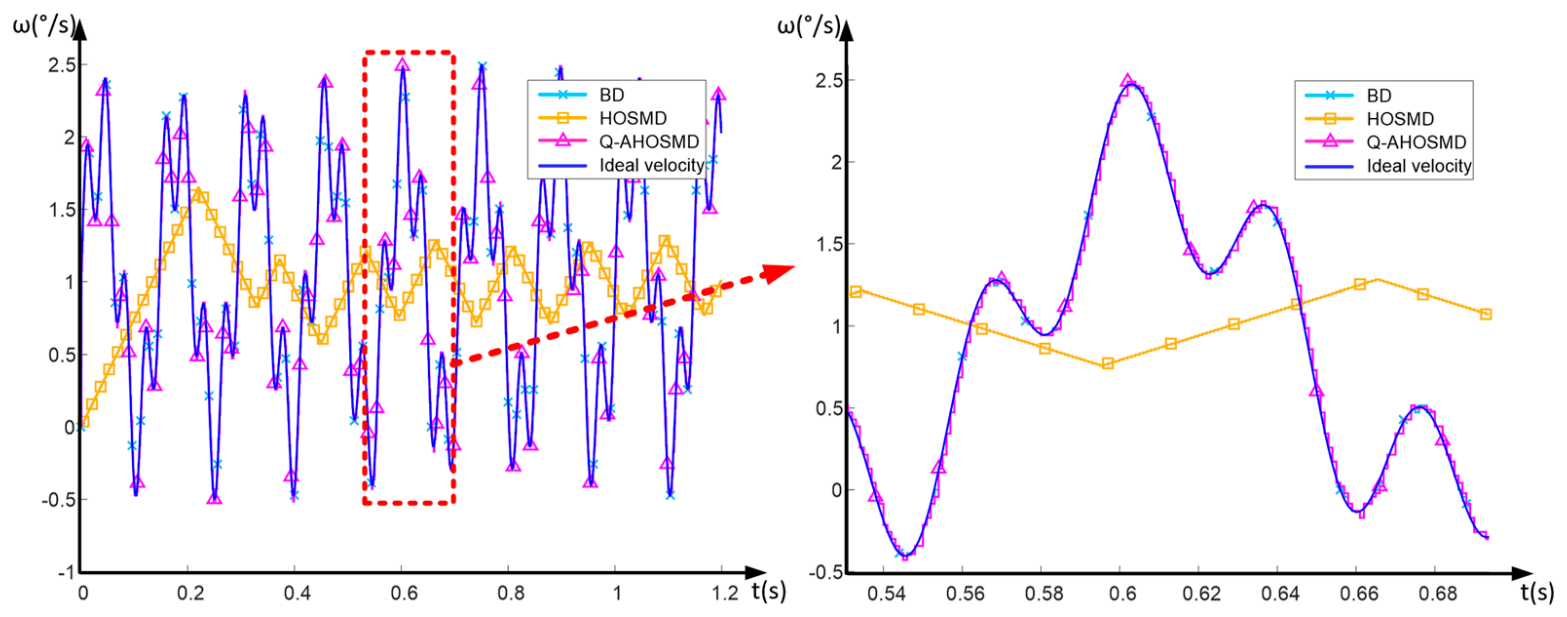
Velocity estimation curves under the higher-speed sinusoidal condition.

With the true angular position input *f*(*t*) = (1 + sin(2*π* × 7*t*) + 0.5sin(2*π* × 27*t*))°, the velocity estimation curves and the error statistics are shown in Figure 8 and Table 4.

It can be seen that under the higher-speed condition, the influence of the quantization error on BD velocity estimation is diluted, and the BD can reconstruct the velocity variation from the position information relatively accurately; because *L* is too small, the HOSMD cannot track the larger acceleration under this condition, and its error increases to 0.91°/s. The RMS error of the Q-AHOSMD (0.018°/s) is comparable to that of the BD (0.017°/s), indicating that the performance of the proposed method does not degrade under non-quantization-limited conditions.

## 6. Conclusions

Focusing on the information sparsity problem of quantized position signals under ultra-low-speed conditions, this paper proposed the Q-AHOSMD and established a complete convergence theory. The main conclusions are as follows:

(1) The saturation switching mechanism with the quantization resolution as prior knowledge, the event-driven decaying differential velocity estimation, and the velocity-mismatch-feedback adaptive gain cooperate to endow the differentiator with both smoothness and rapid convergence without relying on a dynamic model.
(2) Finite-time convergence in the noise-free case and practical stability under quantization noise are proved theoretically; the ultimate bounds are explicitly available, shrink continuously with the quantization unit, and achieve the optimal asymptotic order of exact differentiators.
(3) A set of engineering tuning guidelines requiring only sensor specification parameters was formed. Simulations show that even under the extreme condition of 0.001°/s, the steady-state RMS velocity estimation error of the proposed method still reaches 0.00036°/s, about 94% lower than that of the BD method.

The method is applicable to ultra-precision motion control systems requiring high-quality ultra-low-speed velocity feedback, such as precision turntables, optical tracking mounts, and semiconductor equipment. Future work will proceed in the following directions: combining motor control signals to further improve velocity estimation performance at ultra-low speeds without relying on a model and integrating with ultra-low-speed servo control methods, with experimental verification on a physical precision servo turntable platform.

## Figures and Tables

**Table 1:** Parameters of the HOSMD and the Q-AHOSMD.

Differentiator	Parameters
HOSMD	*λ*_0_ = 1.1, *λ*_1_ = 1.5, *L* = 5
Q-AHOSMD	*λ*_0_ = 1.1, *λ*_1_ = 1.5, *κ* = 1/2, *p* =1.5, *L_min_* = 0.001

**Table 2:** Velocity estimation errors (RMS, °/s) under the ultra-low-speed constant-velocity condition.

Condition	Velocity Estimation Errors (RMS, °/s)		
	BD	HOSMD	Q-AHOSMD
0.001°/s constant velocity	0.0066	0.0064	0.00036

**Table 3:** Velocity estimation errors (RMS, °/s) under the ultra-low-speed sinusoidal condition.

Condition	Velocity Estimation Errors (RMS, °/s)		
	BD	HOSMD	Q-AHOSMD
Ultra-low-speed sinusoidal motion	0.017	0.0070	0.0037

**Table 4:** Velocity estimation errors (RMS, °/s) under the higher-speed sinusoidal condition.

Condition	Velocity Estimation Errors (RMS, °/s)		
	BD	HOSMD	Q-AHOSMD
Higher-speed sinusoidal motion	0.017	0.91	0.018

## Data Availability

The data presented in this study are available from the corresponding author upon reasonable request.
